# Age-Specific Signatures of Glioblastoma at the Genomic, Genetic, and Epigenetic Levels

**DOI:** 10.1371/journal.pone.0062982

**Published:** 2013-04-29

**Authors:** Serdar Bozdag, Aiguo Li, Gregory Riddick, Yuri Kotliarov, Mehmet Baysan, Fabio M. Iwamoto, Margaret C. Cam, Svetlana Kotliarova, Howard A. Fine

**Affiliations:** 1 Neuro-Oncology Branch, National Cancer Institute, National Institute of Neurological Disorders and Stroke, National Institutes of Health, Bethesda, Maryland, United States of America; 2 Department of Mathematics, Statistics, and Computer Science, Marquette University, Milwaukee, Wisconsin, United States of America; 3 Center for Human Immunology, Autoimmunity and Inflammation, National Heart Lung and Blood Institute, National Institutes of Health, Bethesda, Maryland, United States of America; 4 The Neurological Institute of New York, College of Physicians and Surgeons, Columbia University, New York, New York, United States of America; 5 New York University Cancer Institute, New York University Langone Medical Center, New York, New York, United States of America; Wake Forest University, United States of America

## Abstract

Age is a powerful predictor of survival in glioblastoma multiforme (GBM) yet the biological basis for the difference in clinical outcome is mostly unknown. Discovering genes and pathways that would explain age-specific survival difference could generate opportunities for novel therapeutics for GBM. Here we have integrated gene expression, exon expression, microRNA expression, copy number alteration, SNP, whole exome sequence, and DNA methylation data sets of a cohort of GBM patients in The Cancer Genome Atlas (TCGA) project to discover age-specific signatures at the transcriptional, genetic, and epigenetic levels and validated our findings on the REMBRANDT data set. We found major age-specific signatures at all levels including age-specific hypermethylation in polycomb group protein target genes and the upregulation of angiogenesis-related genes in older GBMs. These age-specific differences in GBM, which are independent of molecular subtypes, may in part explain the preferential effects of anti-angiogenic agents in older GBM and pave the way to a better understanding of the unique biology and clinical behavior of older versus younger GBMs.

## Introduction

Glioblastoma multiforme (GBM) is the most common malignant primary brain tumor [1]. GBM patients have a median survival of about fourteen months despite aggressive multimodality treatment [2]. Given the pathological and clinical heterogeneous nature of GBMs, there have been a number of recent attempts to better understand and characterize these tumors at the molecular and genetic level [3]–[16]. Among these studies, The Cancer Genome Atlas Project (TCGA) has generated a vast amount of high-throughput data for about 500 GBM samples [4], [15].

Advanced age has been identified as an independent significant prognostic factor for survival in glioblastoma clinical trials since the 1970s (Table S1). An analysis of three randomized phase III trials conducted by the Radiation Therapy Oncology Group (RTOG) showed that median survival of GBM patients aged 60 or older was 7.5 months compared to 16.2 months in patients younger than 40 years old [17]. Older age as negative prognostic factor for GBM survival was confirmed by other National Cancer Institute sponsored cooperative groups [18], [19] and a large meta-analysis of 3,004 patients with high-grade gliomas [20]. The study that established the current standard of care for newly diagnosed glioblastoma with radiation and concurrent temozolomide also showed a shorter median survival of patients older than 60 years (11.4 months) compared to those who were 50 years or younger (17.4 months) [21].

The reasons why older age is such a negative prognostic factor remain unclear. Retrospective data and randomized controlled trials do not suggest that older patients receive less than optimal treatment and/or tolerate treatment less well than younger patients thereby suggesting a potential difference in the biology of GBMs in older patients. Thus, it would be valuable to discover age-specific signatures in GBM biology that might explain this survival difference and allow clinicians to develop age-specific therapeutic clinical trials for GBM.

Noushmehr, *et al*. discovered a glioma-CpG island methylator phenotype (G-CIMP) in GBMs [22]. G-CIMP positive patients (about 11% of GBM samples in TCGA) have significantly longer survival than G-CIMP negative patients. G-CIMP positive patients are also significantly younger than G-CIMP negative patients. Nevertheless, age still turns out to be a significant independent prognostics factor for survival despite the G-CIMP status of the tumor [22].

In this study, we computationally analyzed gene expression, exon expression, microRNA expression, DNA methylation, copy number alteration, somatic mutation derived from whole exome sequence, and SNP data sets of the TCGA GBM samples to discover age-specific signatures at the transcriptional, genetic, and epigenetic levels. In order to avoid the confounding variable of the G-CIMP status of the tumor, we trained a model to predict G-CIMP status of GBM samples based on gene and exon expression profiles in order to exclude G-CIMP positive patients in our analyses.

## Materials and Methods

### Determining Old and Young GBM Groups

For two-sample tests, we defined a “young” and “old” group. We hypothesized that if there is an old and young biology then samples with “intermediate” ages might represent a mix of these biologies. Thus, we did not include samples with “intermediate” ages in our old and young groups. In order to define the age boundaries for the old and young groups, we examined the histogram of survival for different age groups (Figure S1) and number of samples in each age group (Figure S2). We assigned patients ≤40 years old to the *young* group and patients ≥70 years old to the *old* group. The number of available samples in the young and old groups changes depending on the data set (Table 1), but overall young and old patients constitute 9% and 21% of all samples, respectively. For linear regression tests, we also used samples with *intermediate* ages (i.e., between 40 and 70) to increase the power of the analysis.

**Table 1:** Number of GBM samples used in this study (downloaded from the TCGA repository on June 29, 2011, Sample IDs are in Table S1).

Data Type	Platform	Level^1^	Institute	# Old^2^	# Young^2^	Total
Gene expression	Affymetrix HT Human GenomeU133 Array Plate Set	2	Broad Institute of MIT and Harvard	92	37	422
Exon expression	Affymetrix Human Exon 1.0 STArray	3	Lawrence Berkeley National Laboratory	80	34	382
Gene expression	Agilent 244K Custom GeneExpression G4502A	2	University of North Carolina	92	37	420
miRNA expression	Agilent 8×15K HumanmiRNA-specific microarray	3	University of North Carolina	80	34	385
Methylation	Illumina Infinium Human DNAMethylation 27	2	Johns Hopkins/University of Southern California	56	22	256
Copy Number	Agilent Human Genome CGHMicroarray 244A	3	Memorial Sloan-KetteringCancer Center	87	36	406
SNP	Affymetrix Genome-WideHuman SNP Array 6.0	3	Broad Institute of MIT and Harvard	88	32	390
SNP	Illumina 550K InfiniumHumanHap550 SNP Chip	3	HudsonAlpha Institute for Biotechnology	78	33	376
Whole ExomeSequence	Illumina Genome Analyzer IIx	N/A	Broad Institute of MIT and Harvard	55	12	202

1 Level 2 refers to probeset-level data and level 3 refers to gene-level data for expression and methylation data sets. Level 3 refers to segmented data for copy number and SNP data sets. There is no level number for whole exome sequence data set as we just used the mutations derived from this data set.

2 Old and Young refer to samples ≥70 and ≤40 years old, respectively.

### Predicting G-CIMP Status of GBM Patients and Removing G-CIMP Positive Patients

**Figure 1:**
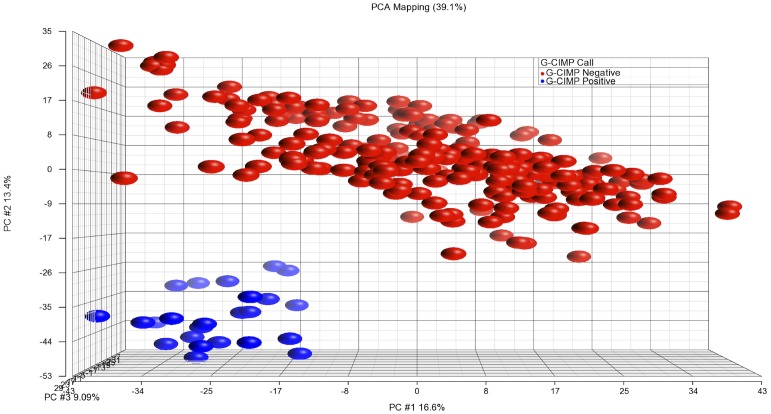
PCA plot of GBM samples with methylation data. Red: G-CIMP negative, Blue: G-CIMP positive. Methylation sites with std. deviation >0.2 are selected to generate this graph.

We obtained G-CIMP calls of samples that have methylation data from [22] (Figure 1). To predict G-CIMP calls of samples for which no methylation data is available, we used the k-nearest neighbor algorithm to train models from gene and exon expression profiles of samples with a G-CIMP call. The G-CIMP call prediction results from models of gene and exon expression overlapped by more than 95%. We chose consistent prediction calls as final G-CIMP calls. All analyses were conducted on Partek® Genomics SuiteTM version 6.5 (Copyright © 2010 Partek Inc., St. Louis, MO, USA).

G-CIMP positive patients are significantly younger than G-CIMP negative patients and there is a significant difference between G-CIMP positive and negative patients at the transcriptional, genetic, and epigenetic levels [22]. If we compared the old and young groups without eliminating G-CIMP positive samples, some of the G-CIMP-specific signatures would be potentially considered as age-specific (i.e., Type I error). In order to eliminate this error, we excluded the G-CIMP positive samples, which constituted about 11% of the database in our analysis.

### Computing Age-specific Differentially Expressed/methylated Genes and microRNAs

We used data sets from Affymetrix U133A, Affymetrix Human Exon 1.0, and Agilent 244 K G4502A platforms in TCGA for differential gene expression analysis (Table 1). We used DNA methylation data set from Illimuna Infinium Human DNA Methylation 27 in TCGA for differential methylation analysis (Table 1). The sample IDs for each data set are listed in Table S2. We used data set from Agilent 8×15 K Human miRNA-specific microarray in TCGA for differential microRNA expression analysis (Table 1). Finally, we used 100 microRNA-specific probes in the Illumina Infinium platform for differential microRNA methylation analysis. We applied two-sample t-test to compute age-specific differentially expressed genes (DEGs) between the old and young groups. Considering age as a continuous variable, we applied linear regression to compute age-specific DEGs. We also applied a nonparametric *ranked-based* linear regression to find age-specific differentially expressed microRNAs and differentially methylated genes (DMGs). We used a ranked-based linear regression on microRNA expression and DNA methylation data, since these data were not normally distributed. We used the samr v1.28 package in R [23] for all tests. For multiple test correction, we applied a permutation-based FDR threshold of 0.05.

### Computing Age-specific Differentially Altered Genes

We used segmented copy number/SNP data sets in Agilent HG CGH 244 K, Affymetrix Human SNP 6.0, and Illumina 550 K Infinium HumanHap550 platforms in TCGA (Table 1). The samples from Agilent HG CGH 244 K data set cover all samples in Infinium HumanHap550 data set and 95% of the samples in SNP6 data set (Figure S3). We performed the analysis on each data set independently. We generated a project in Nexus v5.1 (BioDiscovery Inc., El Segundo, CA, USA) for each data set and used Nexus’ *comparison* function to find differentially altered regions between old and young groups (q-value≤0.05). The *comparison* function compares the frequency of alteration in both groups and finds areas where there is significant difference in frequency [24].

### Somatic Mutation Data Analysis

We used somatic mutations derived from whole exome sequences in TCGA (TCGA Analysis Working Group Data Release Package 1, 8/26/2011). We performed Fisher’s exact test to find genes that are significantly mutated in old or young GBMs.

### Survival Analysis

We applied Cox multivariate analysis on variables namely age, molecular subtypes derived in [15] (i.e., classical, neural, mesenchymal, proneural), gender, and Karnofsky performance score. We used *coxph* function in R [25]. We also generated Kaplan-Meier survival plots in Partek® Genomics Suite™ version 6.6.

### Functional Analysis

We applied the gene set enrichment analysis (GSEA) algorithm [26], [27] to identify upregulated expression pathways and signatures by comparing old and young groups. GSEA mapped all 3272 gene sets in the functional c2 v3 MsigDB database to ranked genome-wide expression profiles (Affymetrix U133A) of old versus young groups. To compute p-values for enrichment scores, we applied Kolmogorov-Smirnov statistics by constructing a cumulative null distribution with permuting old and young group assignments 1000 times. The significant gene sets were claimed for nominal *p*≤0.05. We also used DAVID [28] to create functional annotation charts on age-specific upregulated genes derived from both Affymetrix U133A and Agilent 244 K G4502A data sets via a linear regression method (Fisher’s exact test p≤0.05) and Ingenuity Pathway Analysis (IPA) software (Ingenuity® Systems, www.ingenuity.com) on age-specific angiogenesis related genes to display the interactions among these genes.

### Motif Enrichment Analysis

In order to compute enriched motifs in the promoters of the age-specific DEGs, we used PScan [29] to find enriched motifs in the JASPAR database [30]. We checked sequences from 450 bp upstream to 50 bp downstream of the transcription start size for each Refseq transcript of the gene in human genome version hg19. We applied a Benjamini-Hochberg multiple test correction method [31] to correct for multiple testing.

### Cross-validation on TCGA Data Set

To validate age-specific DEGs, we applied 10-fold cross-validation on TCGA U133A data set of old and young patients. For each fold, we used the support vector machine (SVM) algorithm to build a model based on training data (i.e., age-specific DEGs obtained from 90% of samples) and used this model to predict the old/young status of the remaining 10% of samples. For comparison, we also used the same algorithm to build a model based on the molecular subtype-specific DEGs of training data. We used Partek® Genomics SuiteTM version 6.5 and tried different parameter choices for the SVM algorithm. To compute DEGs, we used one-way ANOVA.

### Validation on External Data Set

To validate the age-specific signatures derived from the TCGA data set, we obtained gene expression profiles of GBM samples from the REMBRANDT database (http://caintegrator-info.nci.nih.gov/rembrandt). We predicted the G-CIMP status of these samples as described above. There were 153 G-CIMP negative samples (27 old, 15 young, and 111 intermediate). Due to the small sample size, we were unable to compute statistically significant age-specific DEGs on this data set. We, therefore, filtered in the age-specific genes at the transcriptional level derived from both TCGA Agilent 244 K G4502A and Affymetrix U133A data sets (hereafter the TCGA age signature) in the REMBRANDT data set to create a *filtered* data set. We clustered the filtered data set via hierarchical clustering to see if old and young samples would be separated by TCGA age signature. We also clustered the unfiltered REMBRANDT data set (i.e., all genes) and compared both results. As a more quantitative approach, we also built an ANOVA model on filtered and unfiltered REMBRANDT data sets to compute how much of the variation could be explained by the age group (i.e., old and young). We built a 3-way ANOVA by using gender, age group, and sample source institute as categorical factors. We used Partek® Genomics Suite™ version 6.6 for clustering and building ANOVA model.

## Results

### Age is an Independent Significant Prognostic Factor for Survival within G-CIMP Negative GBMs

**Figure 2:**
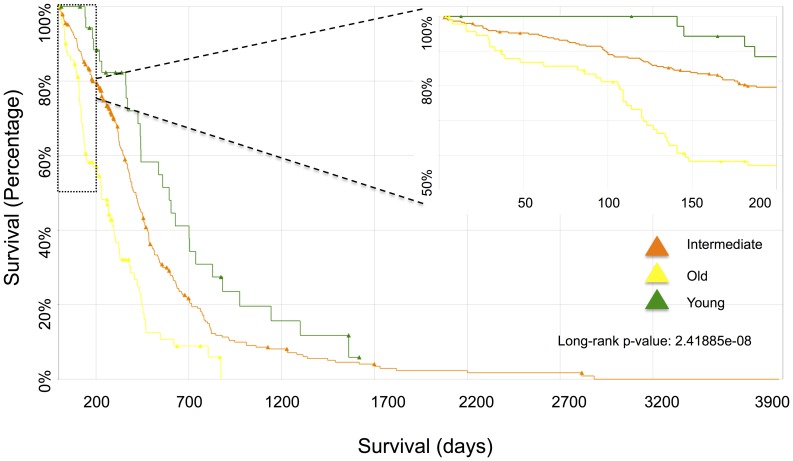
Kaplan-Meier plot between old, young, and middle-aged GBM samples.

Age is known to be an independent significant prognostic factor for survival in GBMs [1], [22]. Our multivariate Cox regression analysis on G-CIMP negative GBM samples also demonstrated that age is an independent significant factor for survival within G-CIMP negative GBM patients (p-value<5.02e-07, Table S3). The Kaplan-Meier plot also shows that there is significant survival difference between old and young GBM samples (log-rank p≤2.42e-08, Figure 2). Of note, our results show that GBM molecular subtypes are not a significant factor for survival as previously reported [15].

### Age-specific Signature at the Transcriptional Level

We computed age-specific DEGs by using both t-test and linear regression. DEGs found by linear regression mostly contain DEGs found by t-test in all three platforms suggesting that the use of all samples gives more power to the analysis (Table 2). Using linear regression, we found 1749, 909, and 91 DEGs in Affymetrix U133A, Agilent G4502A, and Affymetrix Human Exon 1.0 platforms, respectively (FDR<0.05, Table S4). The low number of DEGs in the exon data set is possibly due to the lower sample size compared with the other data sets. There are 334 DEGs found both in Agilent G4502A and Affymetrix U133A platforms. Among these DEGs, seven of them (*SOD2, GTPBP4, TPST1, GNA12, SIM2, ZFP2, and SLC22A5*) have also age-specific differential expression in normal brain tissues based on a data set described in [32]. Two hundred and thirty of these genes (69%) are upregulated in older GBMs. There are fourteen genes that were found by both tests in all three platforms (upregulated in old: PRUNE2, TMEM144, SLC14A1; downregulated in old: H2AFY2, ENOSF1, SFRP1, RANBP17, SVIL, TUSC3, ATF7IP2, FZD6, TSPYL5, DLK1, HIST3H2A). A number of these genes are of apparent interest for GBM biology such as TUSC3, which is a tumor suppressor candidate gene and known to be hypermethylated in GBMs [33]. Additionally, SFRP1 and FZD6 are in the Wnt signalling pathway [34].

**Table 2:** Number of differentially expressed genes between Old and Young GBM samples for three transcriptomic platforms.

	T-test^1^	Linearregression^1^	Common
Affymetrix HT HG U133A	630	1749	**595**
Affymetrix Human Exon 1.0 ST	62	91	**40**
Agilent 244K G4502A	348	909	**313**
**Common (U133A and** **G4502A)**	**130**	**334**	**115**
**Common (all three** **platforms)** 2	**17**	**40**	**14**

The last row shows the number of differentially expressed genes found in all three platforms.

1 In each test, FDR≤0.05 threshold is applied.

2 Shows the number of differentially expressed genes found in all three platforms.

### Age-specific microRNA Expression Signature

We applied ranked-based linear regression and found 19 differentially expressed microRNAs (FDR<0.05) (ebv-miR-BART1-5p, hsa-miR-422b, hsa-miR-507, hsa-miR-147, ebv-miR-BHRF1-2, hsa-miR-620, hsa-miR-554, hsa-miR-625, hsa-miR-661, hcmv-miR-UL70-5p, hsa-miR-325, hsa-miR-453, hsa-miR-552, hsa-miR-558, hsa-miR-223, hsa-miR-302c, hsa-miR-142-5p, hsa-miR-649, hsa-miR-142-3p). All these microRNAs are downregulated in older GBMs. We used the mirWalk database [35] to find experimentally validated targets of these microRNAs. We found 172 experimentally validated target genes (Table S5). Two of these target genes are upregulated in older GBMs (LOX, VEGFA). VEGFA is known to be upregulated in older GBMs [16], [36]. LOX and HIF-1 act in synergy to help tumor cells adapt to hypoxia [37].

### Age-specific Signature at the Epigenetic Level

We found 389 age-specific DMGs by using ranked-based linear regression (Table S6). Ninety-eight percent of these DMGs are hypermethylated in the older GBMs. Seventeen genes that are hypermethylated in the older GBMs are polycomb group protein target genes (PCGTs) (Table S7, Fisher’s exact test p-value<1.0e-10). Hypermethylation of PCGTs has been previously shown to be associated with aging [38]. We subtracted out genes that are normally methylated in an age specific manner based on previous data sets [39], and found 184 and four genes that are uniquely hypermethylated in the old and young GBMs, respectively (Table S8). Eighteen of the GBM-specific DMGs exist in the Pubmeth database [40], which stores genes that are known to undergo methylation in cancer (Table S9, Fisher’s exact test p-value<1.27e-05). Eleven genes are both differentially expressed (Agilent and Affymetrix U133A platforms) and methylated with respect to age (Table S10). Seven of them are hypermethylated and downregulated in older GBMs (MYO1B, PRKCB1, VRK2, FZD6, DLK1, SLC25A21, MSC). We also found three differentially methylated microRNAs (hsa_miR_196b, miR_34b, and miR_34c), all of which were hypermethylated in the old group.

### Age-specific Signature at the Genetic Level

**Figure 3:**
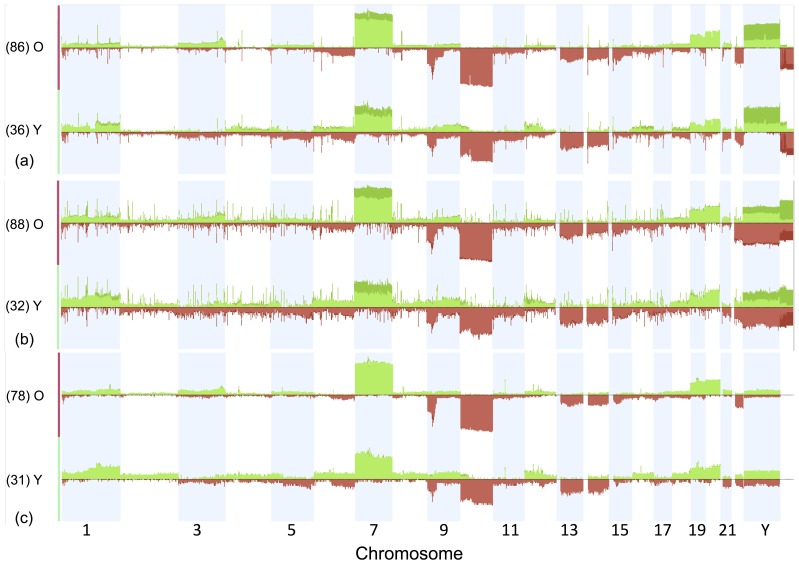
Genome-wide copy number alteration profiles of old and young GBM samples. Data are from (a) Agilent Human Genome CGH Microarray 244A (Memorial Sloan-Kettering Cancer Center), (b) Affymetrix Genome-Wide Human SNP Array 6.0 (Broad Institute of MIT and Harvard), (c) Illumina 550 K Infinium HumanHap550 SNP Chip (HudsonAlpha Institute for Biotechnology) platforms (chr 1–23). Green bars represent amplification and red bars represent deletion. The height of each bar represents the frequency of the alteration in the group. The differentially amplified genes are in chromosome 7 and differentially deleted genes are in chromosome 10.

Each copy number/SNP data set was analyzed in Nexus independently. Figure 3a–c shows the whole genome copy number alteration (CNA) profiles of the old and young groups on these data sets. We found 1044 and 455 differentially altered genes (DAGs) in Affymetrix SNP 6 and HG-CGH 244A platforms, respectively (Table S11). The DAG list found in SNP 6 platform covers 88% of DAGs found in HG-CGH 244A. We could not detect any DAGs on HumanHap550 platform possibly due to the low resolution of this data set. We found the largest DAG list on SNP 6 platform possibly because of its high resolution.

Analyzing the SNP 6 data set, we detected differential deletions only on chromosome 10 for 722 genes. We observed that the old group had a higher frequency of deletion than the young group. We found 321 differentially amplified genes on chromosome 7 with a higher frequency in the old group than the young group and one gene on chromosome 1q (CFHR3) with a higher frequency in the young group than the old group. The high frequency of chromosome 10 deletion and chromosome 7 amplification in the old group, and high frequency of chromosome 1q amplification in the young group have also been reported in a study that compared a cohort of pediatric GBMs with adult GBMs [41].

We compared the list of DAGs on SNP 6 and the list of DMGs, and found three genes that are both heterozygously deleted and hypermethylated in the old group (HHEX, ITGA8, RASGEF1A). Among these genes, HHEX is downregulated in older GBMs. HHEX is known to downregulate VEGF and VEGF receptors [42]. We also observed a significant negative correlation between HHEX and VEGFA expression levels in the TCGA data set (Table S12).

We also compared the list of DAGs on SNP 6 and the list of DEGs found on both Agilent G4502A and Affymetrix U133A data sets. There are 21 genes that are deleted and downregulated, and 7 genes that are amplified and upregulated in the old group (Table S13).

We also analyzed somatic mutations derived from whole exome sequence data. In general, there are more mutations in the old group (Table 3). There are two genes that stand out: TP53 is mutated in 19 old samples and in only one young sample (Fisher’s exact test p-value<0.068). GRM3 is mutated in 3 out of 12 young samples and none of the old samples (Fisher’s exact test p-value<0.01645).

**Table 3:** Number of mutated genes in old and young GNEG GBMs.

Number of samples^1^	Number of mutated genes in Old	Number of mutated genes in Young
>0	3038	720
>1	561	37
>2	159	3 (GRM3, TTN, PTEN)
>3	49	0
>6	8 (PTEN, EGFR, MUC16, TTN, TP53, RYR2, SLIT3, LRP2)	0
>8	5 (PTEN, EGFR, MUC16, TTN, TP53)	0
>15	2 (PTEN, TP53)	0

1 Shows number of old of young samples each gene is mutated. For instance, there are 3038 genes that are mutated in at least one old sample (see first row) and PTEN is mutated in more than 15 old samples (see last row).

### Motif Enrichment Analysis

We analyzed the promoter regions of differentially expressed genes appear in Affymetrix U133A and Agilent G4502A data sets. We have found several motifs statistically enriched in the promoter regions of these genes including HIF-1A and MYC (FDR≤0.05 Table S14).

### Functional Analysis of Age-specific DEGs

We ran a GSEA on the old and young groups to discover upregulated gene sets for each group (Table S15). GSEA analysis found that the younger GBMs maintain an active regulation of G1 entry checkpoint in cell cycle (p<0.05) and have a quiescent phenotype (p<0.03). Older GBMs uphold a strong oxygen depletion environment (p<0.04) that induces the hypoxia inducible factor signaling as indicated by three up-regulated HIF signatures (p<0.05) (Table S15). Furthermore, carbohydrate metabolism with over-expressed glycolysis and glucagon signaling reactomes are enhanced in older GBMs. Younger GBMs showed enrichment of P38_MAPK signaling (p<0.01) and upregulated targets of MYC (p<0.04), BMYB signature (p<0.02), and enhanced stem cell signatures (p<0.02) (Table S15). Moreover, a premalignant signature driven by hepatic stem cell marker, epithelial cell adhesion molecular (EpCAM) is enhanced in younger GBMs (p<0.04) whereas older GBMs showed more advanced tumor profiles (p<0.01) and more invasive expression signatures regulated by integrin-mediated cell migration (p<0.03). Additionally, glioblastoma tumor in young patients showed an increased TNF signaling (p<0.05) and protein translational activities (p<0.03), as indicated by the formation of translation initiation complex involving 43S unit (Table S15).

We also ran DAVID on upregulated genes that appear in the Affymetrix U133A and Agilent G4502A data sets. We found enrichment in several GO terms such as “response to hypoxia” (p-value<0.00123, enriched genes: VEGFA, SOD2, BNIP3, SLC11A2, EGLN3, PLOD2, NOL3, and ALDOC); “vasculogenesis” (p-value<0.088, enriched genes: VEGFA, NTRK2, and QKI) (Table S16).

### Cross-validation on TCGA Data Set

We applied 10-fold cross-validation and built a model based on age-specific DEGs on training data and used this model to predict the old/young status of the remaining test samples. The model achieved over 77% prediction accuracy. For comparison, we also used the same algorithm to build a model based on the molecular subtype-specific DEGs of training data. This model predicted about 64% of prediction accuracy.

### Validation on External Data Set

**Figure 4:**
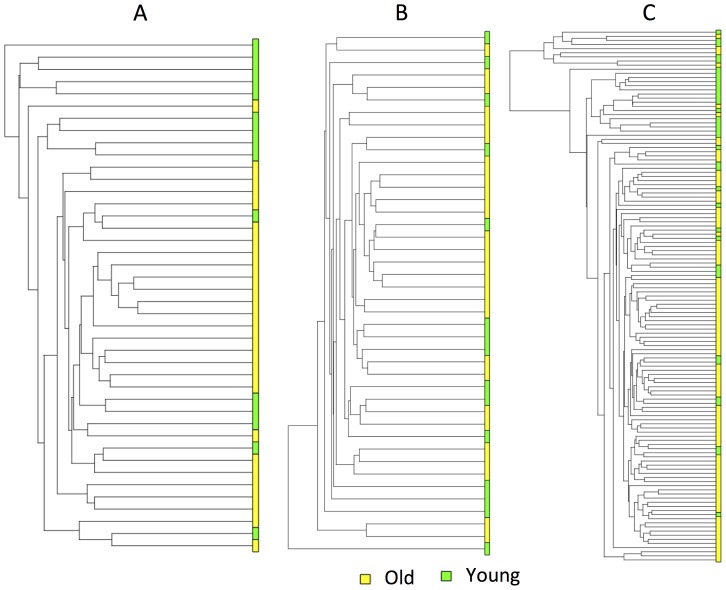
Hierarchical clustering of GBM samples in the REMBRANDT and TCGA data sets. (A) Clustering of old and young REMBRANDT GBM samples based on the expression profiles of age-specific genes derived from both TCGA Affymetrix U133A and Agilent G4502A data sets. (B) Clustering of old and young REMBRANDT GBM samples based on the expression profiles of all genes in the REMBRANDT data set. (C) Clustering of the old and young TCGA GBM samples based on the expression profiles of age-specific genes derived from both TCGA Affymetrix U133A and Agilent G4502A data sets.

**Figure 5:**
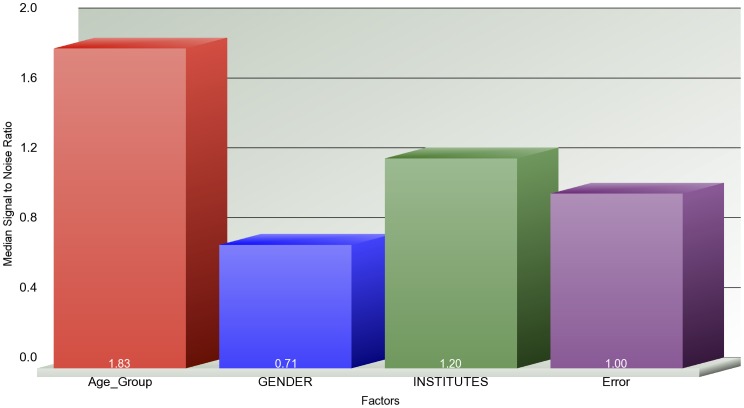
Source of variation of expression profiles of age-specific genes in the REMBRANDT data set. The x-axis shows the components of the 3-way ANOVA model and the y-axis shows the median signal to noise ratio. The ANOVA model is built based on the expression profiles of the TCGA age-specific genes in REMBRANDT data set. The TCGA age-specific genes are the intersection of DEGs computed on TCGA Affymetrix U133A and Agilent G4502A data sets.

**Figure 6:**
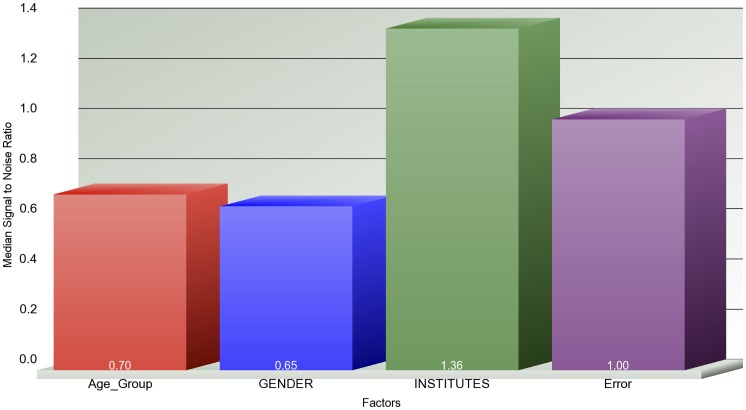
Source of variation of expression profiles of all genes in the REMBRANDT data set. The x-axis shows the components of the 3-way ANOVA model and the y-axis shows the median signal to noise ratio. The ANOVA model is built based on the expression profiles of all genes in REMBRANDT data set.

We obtained the gene expression profiles of G-CIMP negative GBM samples from the REMBRANDT database to validate our findings. We created a filtered REMBRANDT data set by filtering in the TCGA age signature (i.e. age-specific differentially expressed genes derived from both TCGA Affymetrix U133A and Agilent 244 K G4502A data sets via linear regression). We clustered both filtered (Figure 4-A) and unfiltered (i.e. all genes) REMBRANDT data sets (Figure 4-B). To create a reference point, we also clustered old and young GBMs in TCGA Affymetrix U133A data set based on the TCGA age signature (Figure 4-C). We observed that the separation between old and young groups is more apparent in the cluster on filtered data set than the separation on the unfiltered data set (Figure 4). We also observed that the separation of old and young samples in the clustering of filtered data set are very similar to the separation of old and young samples in TCGA Affymetrix data set (Figure 4). Additionally, we built a 3-way ANOVA model on both filtered and unfiltered REMBRANDT data sets by using the categorical factors of age group (i.e., old and young), gender, sample source institute to compute how much of the variation in gene expression is explained by each factor (Figure 5, 6). The results show that age group explains the majority of the variation in the filtered data set, whereas it could not explain the variation in the unfiltered data set.

We also checked whether TCGA DEGs have the same direction of regulation (up or down) in REMBRANDT data set. We applied age-specific linear regression to compute p-value for TCGA DEGs in REMBRANDT data set and created a gene list for both FDR<0.05 and unadjusted p-value<0.05 thresholds. There were 55 and 148 genes in these gene lists, respectively, which had 100% consistency with respect to the directionality of regulation in TCGA and REMBRANDT data sets.

## Discussion

Age has consistently been shown to be one of the most powerful prognostic factors for survival in patients with malignant gliomas with younger patients generally living much longer than older patients. The negative effects of age seen in a number of systemic cancers have often been ascribed to the physiological stress of metastatic cancer in the setting of concurrent medical problems leading to an increased rate of medical related deaths [43]. Additionally, the poor tolerance of older patients to aggressive toxic systemic chemotherapy often results in either treatment-related complications and/or suboptimal tumor treatment [44]. These clinical variables do not, however, adequately explain the profoundly negative effect of age in patients with GBM since such patients almost never have metastatic disease and do not usually die of concurrent medical problems. Furthermore, the marginal effects of systemic chemotherapy in patients with GBMs means that patients are generally treated less aggressively than patients with systemic cancer and the amount of chemotherapy that GBM patients receive has little impact on overall survival. Furthermore, there are few data to suggest that involved field radiotherapy, the one effective treatment for GBM, is associated with increased mortality in older versus younger patients. The lack of a clinical explanation for the poorer survival of older patients with GBM, together with the growing appreciation of the heterogeneous nature of the disease, leads to the hypothesis that the impact of age on survival may be do to a difference in the biology of GBMs in older patients compared to that in younger patients.

There have been a relatively large number of studies over the last decade demonstrating that GBM is a heterogeneous tumor with the most recent studies suggesting that there are at least four major molecular subtypes of GBM based on gene expression profiling [15]. Those major subtypes, however, do not account for the effect of age on survival. Recently, however, the G-CIMP positive subgroup of the proneural subtype of GBM was described based on a pattern of differential genomic methylation [22]. G-CIMP positive GBM patients tend to be younger and have a significantly longer survival than the G-CIMP negative GBMs thus accounting for some of the age-associated effects on survival [22]. Nevertheless, our data demonstrates that age remains a powerful predictor of survival amongst G-CIMP negative tumors. Thus, we sought to elucidate a biological basis for this age–related effect. To do so, we integrated the high-throughput transcriptomic, genetic, and epigenetic profiles of about 425 GBM samples in the TCGA project to find age-specific signatures at the transcriptional, genetic, and epigenetic levels and found such differences at all levels.

We observed a relative small number of DAGs that consistently differentiated old versus young GBMs. Specifically we found that chromosome 10 deletion and chromosome 7 amplification was found commonly in the old group whereas there was a relatively high frequency of chromosome 1q amplification in the young group, observations that have been previously reported in a study that compared a cohort of pediatric GBMs with adult GBMs [41].

In contrast to the relatively few consistent genomic changes between each group of tumors, we did observed a large age-specific signature at the transcriptional level. We observed a major overlap between the DEGs found in Affymetrix U133A and Agilent G4502A platforms, although not surprisingly, each platform had unique DEGs as described in [45]. We observed fewer DEGs on the Affymetrix Human Exon 1.0 platform possible due to the lower sample size in this platform.

We applied 10-fold cross validation on TCGA U133A data set to validate age-specific DEGs. We applied the SVM algorithm to build a model based on age-specific DEGs of training samples and applied this model to predict old/young status of the test samples. This model achieved over 77% prediction accuracy whereas a model based on molecular subtype-specific DEGs only achieved 64% accuracy.

We also validated age-specific signatures at the transcriptional level on an external data set. We obtained G-CIMP negative old and young GBM samples from the REMBRANDT database and clustered these samples based on the TCGA age signature genes and all genes. The clustering results showed that the TCGA age signature could separate the old and young REMBRANDT samples as well as it separates the old and young TCGA samples. We also showed that when the TCGA age signature was selected in the REMBRANDT data set, the majority of the variation could be explained by age group (i.e., old and young). The age group, however, could not explain the variation on the entire REMBRANDT data set. We also showed that the upregulated (downregulated) TCGA DEGs are also upregulated (downregulated) in REMBRANDT data set. These findings indicate that the TCGA age-specific signature at the transcriptional level is also age-specific in REMBRANDT data set.

We also observed a large age-specific signature at the epigenetic level. In particular, we found that about 98% of the DMGs between the old and young group are hypermethylated in the old group. There are several studies that show that aging increases methylation of DNA including cancer-related genes [38], [46]–[48]. The hypermethylated genes in the old group enriched for PCGTs that are known to be undergo methylation with aging [38]. It has been also shown that PCGTs in stem cells undergo hypermethylation with aging and this methylation locks the cell in an undifferentiated state. Thus, our results are consistent with the cancer stem cell hypothesis of gliomagenesis being most prevalent in the older GBMs, in part through hypermethylation of PCGTs. Obviously, such a hypothesis awaits further experimental validation, as do the significance and meaning of our other age-specific epigenetic signatures.

The primary focus of this study was to identify the significant genomic, genetic and epigenetic signatures between young and old GBMs for hypothesis generation and future study and although the annotation and the biological significance of these changes are well beyond the scope of this manuscript, there is one striking observation worth noting. We found a significant number of genes involved in the hypoxic response and in angiogenesis deregulated in older GBMs compared to younger GBMs. In particular, we found a remarkable number of genes involved in the regulation of the proangiogenic protein, VEGF, deregulated in the older GBMs. This is consistent with older pathology-based studies that have demonstrated VEGF to be more highly expressed in older GBMs than in younger GBMs. Examples of the genes we found deregulated at the expression level or through transcriptional factor motif activation in older GBM that contribute to VEGF expression include HIF, HHEX, EGR1, CTCF, HTATIP2, lox, and DLK1.

**Figure 7:**
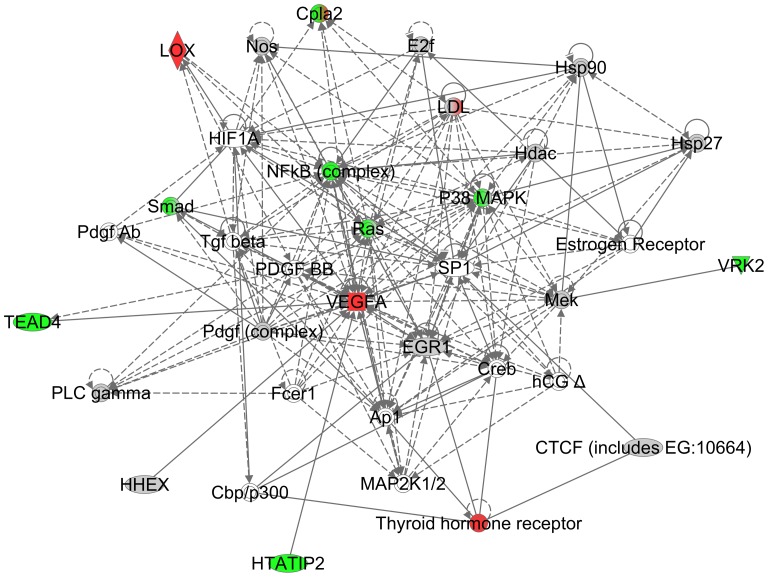
IPA network of angiogenesis-related genes. Node color represents the expression status based on Affymetrix U133A data set (Red: Upregulated in old GBMs, Green: Downregulated in old GBMs, Gray: Baseline, White: Unknown, Mix of green and red: both upregulated and downregulated genes in the complex).

To better elucidate the potential angiogenesis-related signaling aberrations found in older GBMs, we entered into the IPA network analysis a number of the genes deregulated in older GBMs at either the transcriptional level or at the TF motif enrichment level that have been associated with angiogenesis in the literature. These genes included EGR1 [49], VEGF [50], CTCF [50], Myc [51], Mycn [52], Sp1 [53], MSX1 [54], NDRG2 [55], HTATIP2 [56], VRK2 [57], TEAD4 [58], PKRCB1 [59], HHEX [42], HIF1 [37], DLK1 [60], and Lox [37]. The resulting IPA-generated network (Figure 7) demonstrates a complex network with a number of prototypic deregulated GBM genes (i.e. HIF1A, PDGF, TGF-b, Creb, and HCG) located at key nodes. Most prominently displayed in this network is the central role of VEGF.

Thus, it appears that a key biological difference between older GBMs compared to younger GBMs is the central role of VEGF and angiogenesis signaling. Although we cannot know for certain that the more prominent angiogenic profile of older GBMs is responsible in part for the shorter survival of older patients with these tumors, there is an extensive literature linking greater angiogenic potential with decreased survival in GBMs [61]–[63]. Additionally, the central role of VEGF in the biology of GBMs, as determined by this computational analysis, may in part explain recently published data showing that older GBM patients benefit more from treatment with the VEGF inhibitor, bevacizumab, than do younger patients [36], [64]. These clinical observations have been considered paradoxical because responses to therapy with standard cytotoxic agents were historically always greater in younger GBM patients than in older. Our analysis now gives biological rationale to these previously unexplained clinical results with bevacizumab.

In conclusion, we have demonstrated through computational analyses of high-throughput genomic data from hundreds of tumors that there are substantial and consistent biological differences between GBMs found in older patients compared to those found in younger patients. Although the ultimate biological meaning and clinical significance of many of these findings await experimental validation, it appears clear that the pro-angiogenic phenotype of older GBMs compared to younger GBMs has biological, clinical, and therapeutic significance. This finding demonstrates how computational analysis of high-throughput data of a human tumor can help explain long standing clinical observations and point the way to more rationale therapeutics targeted to a specific biological process in selected patients.

## Supporting Information

Figure S1 **Histogram of survival days of samples for each age bin.**
(TIFF)

Figure S2 **Histogram of number of samples for each age bin.**
(TIFF)

Figure S3 **The comparison of copy number/SNP samples obtained from different institutes in the TCGA project.** MSKCC: Agilent Human Genome CGH Microarray 244A (Memorial Sloan-Kettering Cancer Center), BI: Affymetrix Genome-Wide Human SNP Array 6.0 (Broad Institute of MIT and Harvard), HAIB: Illumina 550 K Infinium HumanHap550 SNP Chip (HudsonAlpha Institute for Biotechnology.
(TIFF)

Table S1 **Survival analysis of GBM in randomized phase trials.**
(XLS)

Table S2 **Sample IDs for each data type used in this study.**
(XLSX)

Table S3 **Cox multivariate analysis results for survival.** P-values≤0.05 are bold faced.
(XLSX)

Table S4 **List of differentially expressed genes found by standard linear regression (FDR≤0.05).**
(XLSX)

Table S5 **Experimentally validated targets of the differentially expressed miRs between the old and young GBMs.**
(XLSX)

Table S6 **List of differentially methylated genes found by ranked-based regression (FDR≤0.05).**
(XLSX)

Table S7 **List of differentially methylated genes that are polycomb group protein target genes.**
(XLSX)

Table S8 **List of genes that undergo hypermethylation only in GBMs.**
(XLSX)

Table S9 **List of differentially methylated genes that have been reported to undergo hypermethylation in cancer in Pubmeth database.**
(XLSX)

Table S10 **List of genes that are differentially expressed (Agilent and Affymetrix U133A platforms) and methylated with respect to age.**
(XLSX)

Table S11 **List of differentially altered genes between Old and Young on different data sets.**
(XLSX)

Table S12 **Pearson and Spearman Rank Correlation between HHEX and VEGFA expression on Affymetrix U133A, Agilent G4502A, and Affymetrix Human Exon platforms.** For all correlations, p-values are significant (p≤0.05)
(XLSX)

Table S13 **List of genes that are (a) deleted and downregulated (b) amplified and upregulated in the old group.** DAGs are computed from SNP 6 platform. DEGs are the intersection of genes found in Affymetrix U133A and Agilent G4502A platforms.
(XLSX)

Table S14 **List of enriched motifs in the promoter regions of the differentially expressed genes appear in both Affymetrix U133A and Agilent G4502A platforms.** FDR(BH): Benjamini-Hochberg FDR value
(XLSX)

Table S15 **GSEA results on old vs. young genes.** NES: normalized enrichment score. Gene sets with negative (positive) enrichment scores are active in young (old) group.
(XLSX)

Table S16 **DAVID analysis results on age-specific upregulated genes in old that appear in Affymetrix U133A and Agilent G4502A data sets.** Fisher's exact test p-value<0.05
(XLS)
