# Supplementary figures and images for: Age-Specific Signatures of Glioblastoma at the Genomic, Genetic, and Epigenetic Levels
Source: PLoS One. 2013 Apr 29;8(4):e62982. doi: 10.1371/journal.pone.0062982 (PMC3639162; doi:10.1371/journal.pone.0062982)

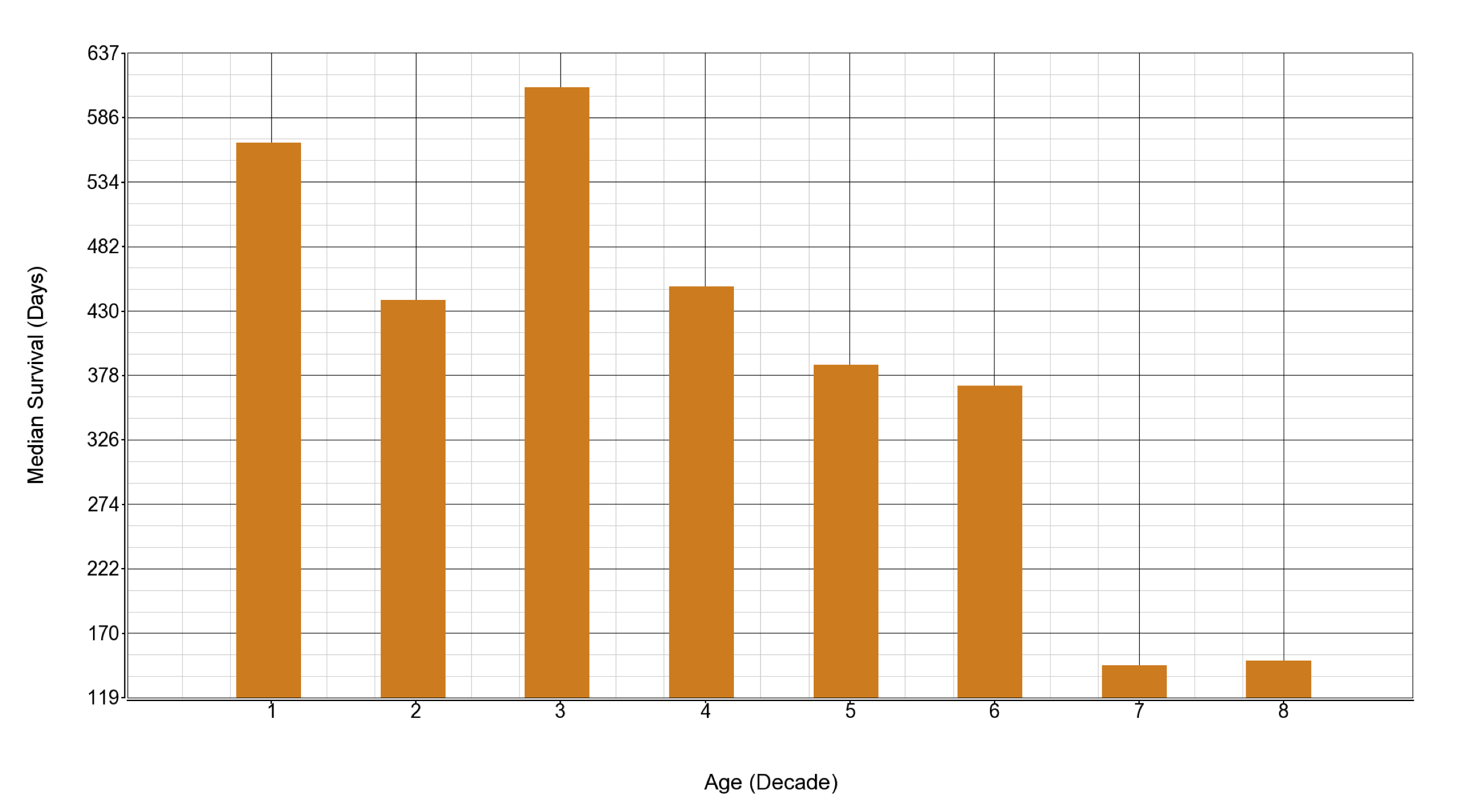

Supplement: Figure S1 — Histogram of survival days of samples for each age bin. (TIFF) [file pone.0062982.s001.tiff]

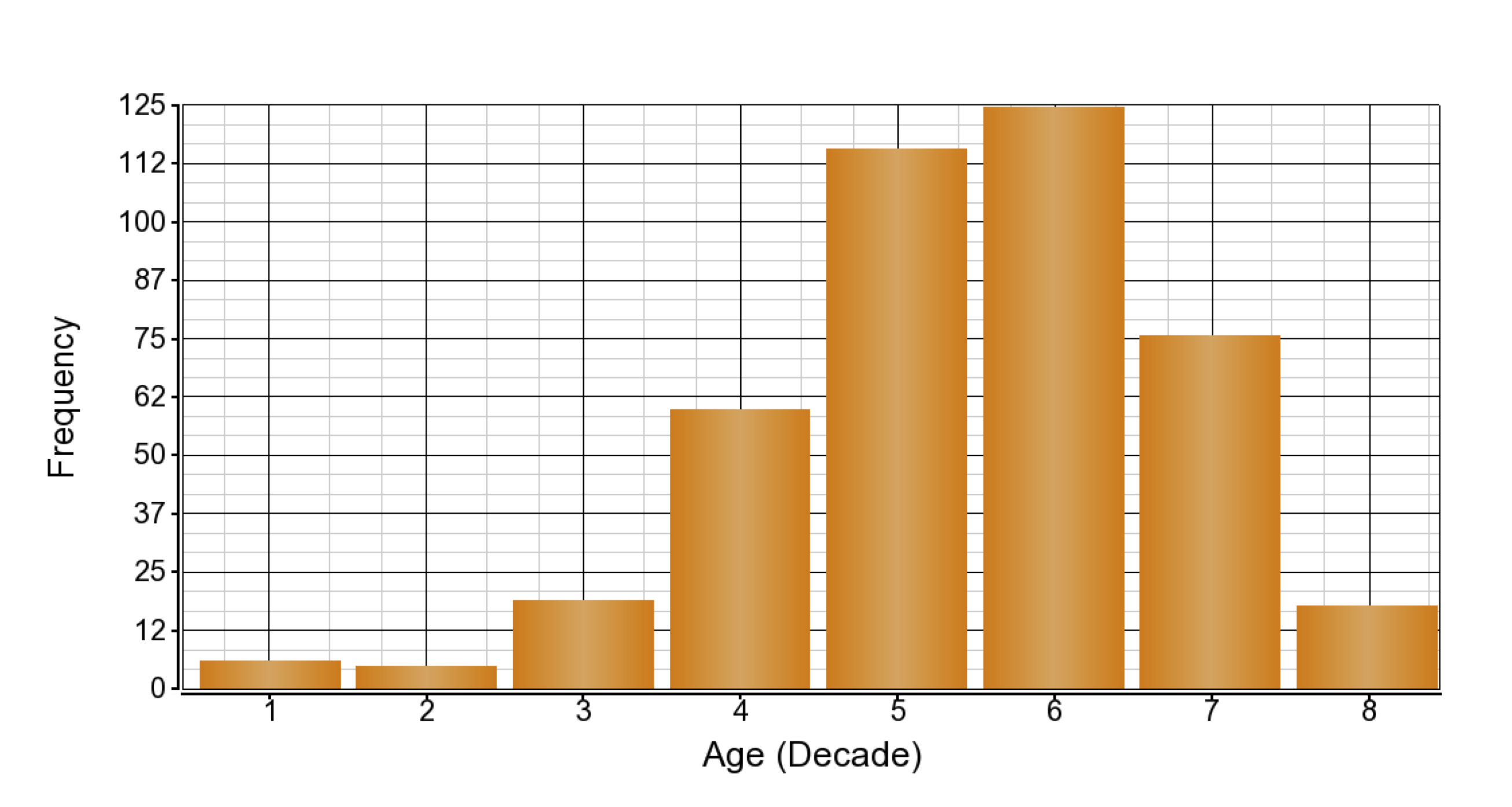

Supplement: Figure S2 — Histogram of number of samples for each age bin. (TIFF) [file pone.0062982.s002.tiff]

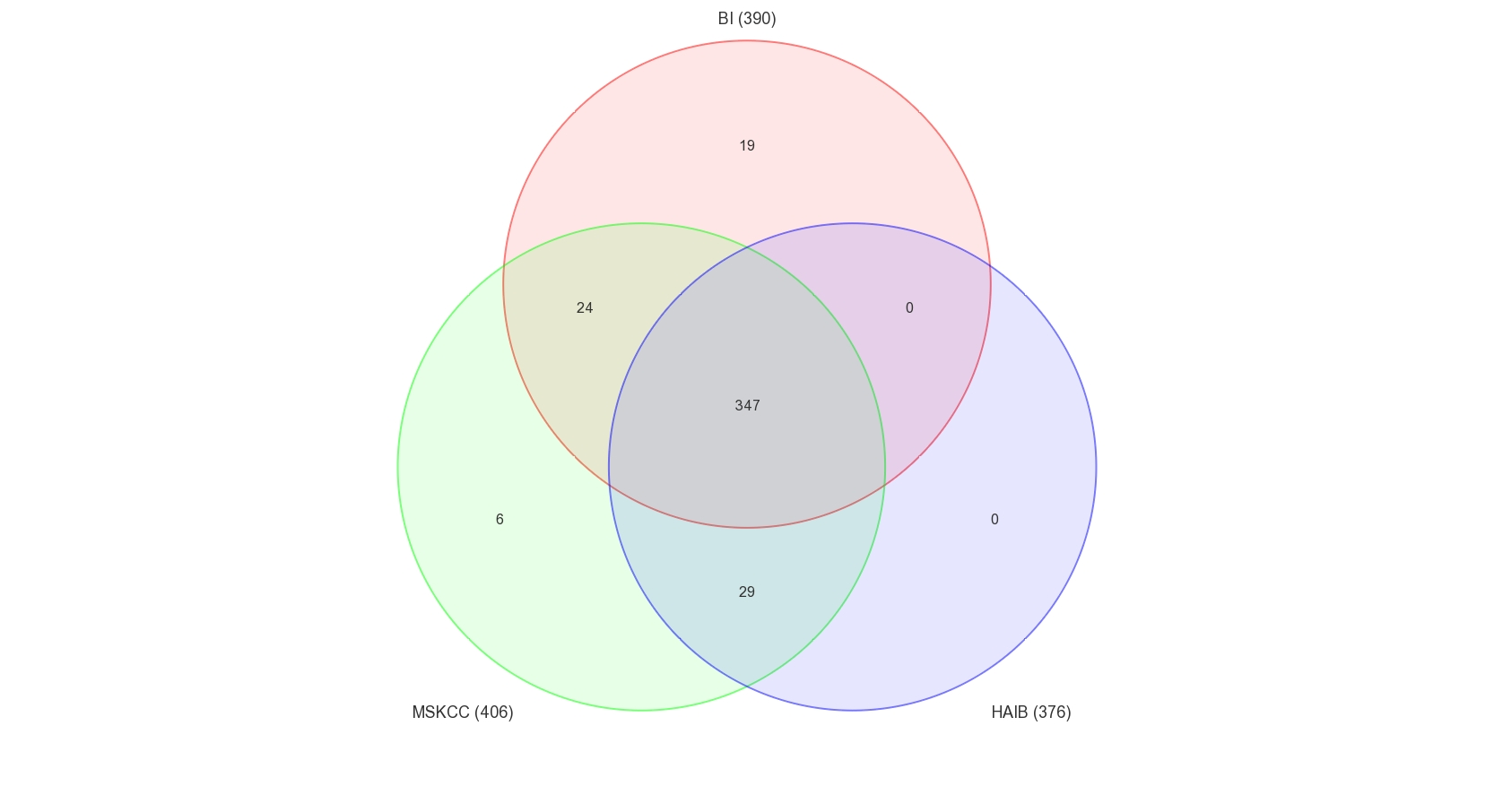

Supplement: Figure S3 — The comparison of copy number/SNP samples obtained from different institutes in the TCGA project. MSKCC: Agilent Human Genome CGH Microarray 244A (Memorial Sloan-Kettering Cancer Center), BI: Affymetrix Genome-Wide Human SNP Array 6.0 (Broad Institute of MIT and Harvard), HAIB: Illumina 550 K Infinium HumanHap550 SNP Chip (HudsonAlpha Institute for Biotechnology. (TIFF) [file pone.0062982.s003.tiff]
